# Detection of Head-to-Tail DNA Sequences of Human Bocavirus in Clinical Samples

**DOI:** 10.1371/journal.pone.0019457

**Published:** 2011-05-04

**Authors:** Jessica Lüsebrink, Verena Schildgen, Ramona Liza Tillmann, Felix Wittleben, Anne Böhmer, Andreas Müller, Oliver Schildgen

**Affiliations:** 1 Institut für Pathologie, Kliniken der Stadt Köln gGmbH, Klinikum der Privaten Universität Witten-Herdecke, Köln (Cologne), Germany; 2 Department of Paediatrics, University Hospital Bonn, Bonn, Germany; Kantonal Hospital St. Gallen, Switzerland

## Abstract

Parvoviruses are single stranded DNA viruses that replicate in a so called “rolling-hairpin” mechanism, a variant of the rolling circle replication known for bacteriophages like ϕX174. The replication intermediates of parvoviruses thus are concatemers of head-to-head or tail-to-tail structure. Surprisingly, in case of the novel human bocavirus, neither head-to-head nor tail-to-tail DNA sequences were detected in clinical isolates; in contrast head-to-tail DNA sequences were identified by PCR and sequencing. Thereby, the head-to-tail sequences were linked by a novel sequence of 54 bp of which 20 bp also occur as conserved structures of the palindromic ends of parvovirus MVC which in turn is a close relative to human bocavirus.

## Introduction

The genus bocavirus belongs to the non-enveloped single-stranded DNA virus family Parvoviridae and consist of three members, bovine parvovirus (BPV), minute virus of canine (MVC), and human bocavirus (HBoV) [1], [2]. As parvoviruses replicate in a so called “rolling-hairpin” mechanism supported by short imperfectly palindromic hairpin telomers their replication intermediates are concatemers of head-to-head or tail-to-tail structure [3]–[6]. However, as to date growing human bocavirus routinely in cell culture failed, the terminal hairpin sequences as well as the replication mechanism remain to be determined; thereby it has to be taken into account that none of the full genome sequences of bocavirus DNA was annotated with the terminal hairpin-like sequences [7], [8]. Taking those observations into account the present study contradicts the conviction that all parvoviruses replicate their genomes by the same mechanism of the rolling-hairpin but that modifications of the rolling hairpin or the rolling circle mechanism are possible for human bocavirus.

The human bocavirus (HBoV), a human parvovirus, was detected in 2005 by Allander and co-workers [1]. In a large series of clinical studies it was associated with respiratory and gastrointestinal infections in all age groups with emphasis in children up to the age of five years ^reviewed by^ [9]–[12]. So far the modified Koch's postulates were not completely fulfilled for HBoV as the virus is frequently detected as a copathogen, is to date not transmittable to small animal models, and was cultured only once in an air-liquid-interface culture with primary human respiratory cells [7], [13]. Thus far it must be assumed that the genome is not deciphered in total as the flanking hairpin structures, a unique but essential feature of parvoviruses, are still unknown. These hairpin-structures are created by short imperfectly palindrome sequences at the viral telomeres [14]–[17]. One distinguishes between heterotelomeric (MVM) and homotelomeric (parvovirus B19) viruses; while heterotelomeric viruses have different terminal sequences, telomeres of homotelomeric sequences consist of inverted terminal repeats [18].

For bovine bocavirus it was shown that it packages up to 90% of the negative strand and that packaging is dependent on so called flip and flop sequences at the terminal structures of the genome [19]. Consequently, but solely based conclusion drawn on phylogenetic analyses, it is hypothesized that HBoV uses such hairpin structures as self-priming elements for its DNA replication, too [13]. Due to the lack of a simple tissue culture model or an animal model, less is known on the replication mechanism of human bocavirus. The genetic map generated by human bocavirus infection supports that HBoV belongs to the genus Bocavirus [7], [20]. In addition, based on the genetic map and on phylogenetic analyses it was assumed – but not shown so far – that HBoV replicates in the typical parvoviral manner [21], the so called rolling hairpin model [3], [14], [22]. In this replication model the replication intermediates are concatemers that have either head-to-head or tail-to-tail structure; in most cases this rolling hairpin replication leads to a more or less equal stochiometry when the progeny genomes are packaged into the newly forming viral particles [21]. For murine parvoviruses it was shown that encapsidation is strongly influenced by the nick site in the viral origin of replication [11], but unfortunately, so far no information is available to which degree this also applies for human bocavirus.

In a previously published study from the authors it was observed that packaged DNA of HBoV in all investigated clinical isolates was of negative polarity and that only a minority of isolates additionally contained the positive strand [23]. These findings led to the hypothesis that HBoV DNA replication or encapsidation may be different from other parvoviruses and also other bocaviruses like MVC. Moreover this observation emphasises the need for mapping the putative flanking hairpin-like structures as such structures are a prerequisite for the postulated rolling-hairpin replication mechanism of HBoV.

## Materials and Methods

### Clinical samples and cell culture supernatant

The study was performed in accordance with an approval from the ethical committee of the University of Bonn (No. 070/06) and in accordance with the declaration of Helsinki in its present form. In order to examine the hypothesis of a modified DNA replication three HBoV isolates from patients suffering from respiratory infections with positive HBoV-PCR and cell culture supernatant from the Bonn-1 strain cultivated in air-liquid-interface cultures were analysed [7]. All material was previously tested positive for human bocavirus DNA by PCR as previously described [24] and was not cleared by centrifugation in advance, thus all materials contained viral particles, cellular debris, but also an unknown amount of cells infected. Furthermore, two high titer isolates were kindly provide from Maria Söderlund-Venermo (Helsinki, Finnland) (Helsinki, Finnland) and Cristiana Nascimento-Carvalho (Salvador, Brazil) and were named Helsinki/Brazil 1 and 2. Thus, the material with the utmost probability contained all forms of viral DNA that do occur during the viral replication cycle.

### Self Priming Elongation Assays

**Figure 1:**
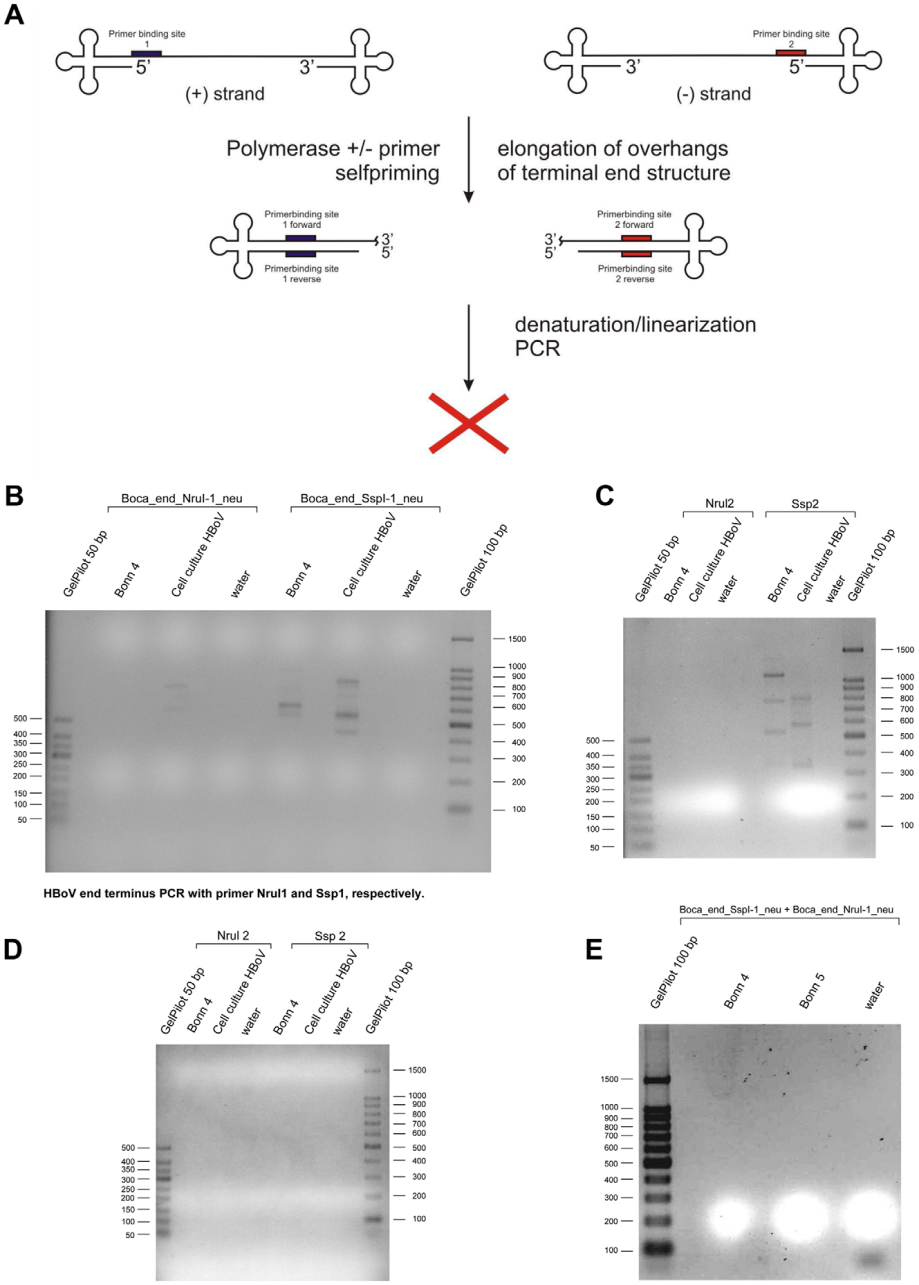
Approach for deciüphering the hairpin like structure of human bocavirus. **a:** ***Putative hairpin structures of human bocavirus.*** In order to test for the self priming capability of the HBoV genome during genome replication different polymerases were added to HBoV DNA isolated from clinical samples. Both, positive strand and negative strand containing isolates were used [23]. Following self-priming elongation the DNA was denaturized and PCR was performed with a single primer. Surprisingly in none of the tested isolates a PCR product was observed, leading to the conclusion that no self priming occurred. **b:** ***HBoV end terminus PCR with primer Nrul1 and Ssp1, respectively.*** HBoV genome preparations were incubated with T7 polymerase and subject to subsequent PCR with primers Boca_end_NruI-1_neu and Boca_end_SspI-1_neu, respectively. Theoretically, elongation of terminal hairpin structures (self-priming) should have occurred as postulated in figure 1a. **c:** ***HBoV end terminus PCR with primer Nrul2 and Ssp2, respectively.*** HBoV genome preparations were incubated with Klenow polymerase and subject to subsequent PCR with primers NruI-2 and SspI-2, respectively. Theoretically, elongation of terminal hairpin structures (self-priming) should have occurred as postulated in figure 1a. **d:** ***HBoV end terminus PCR after circularisation of the genome with primer Nrul2 and Ssp2.*** HBoV genome preparations were incubated with T4 RNA ligase (self-ligation of single strand genomes and subject to subsequent PCR with primers Nru2 and Ssp2, respectively. Theoretically, head-to-tail sequences should have been amplified provided the terminal regions of the genome allow self-ligation and are not masked by secondary structures resistant to self-ligation. **e:** ***HBoV PCR with primer set Boca_end_SspI-1_neu and Boca_end_NruI-1_neu.*** This PCR approach was performed with primer in the outmost terminal region of the HBoV genome DQ000496. The PCR reaction should have amplified head-to-tail, tail-to-tail, or head-to-head structures, provided the target sequence is present in the clinical isolates. Unfortunately the primers did not bind to a terminal region that is know among all so far published isolates, thus it remains unclear whether primer binding was sufficient.

Elongation reactions were carried out according to the scheme presented in figure 1. From HBoV-positive DNA eluates 5 µl were used in a reaction mix with a total volume of 25 µl. This reaction mix contained 1 U T4-DNA polymerase (Invitrogen, Karlsruhe, Germany), 1× T4 DNA polymerase reaction buffer (Invitrogen, Karlsruhe, Germany), 1 mM dNTP mix (Invitrogen, Kalsruhe, Germany), and water. The reaction was carried out at 37°C for 15 min according to the manufacturer's recommendations followed by termination for 15 min at 65°C. No primers were added under the hypothesis that the single stranded HBoV genome should contain terminal hairpin sequences that could exhibit a self priming activity. Following elongation a PCR with a terminal primer was performed (table 1). Thereby, 5 pM primer Head-(SspI) (5′-GGAGGAGTGGTTATATAGA-3′) or Tail-(NruI)(5′-GTGTTACCGTCTCGAACCTAG-3′) or their reverse complement primers were added to 12,5 µl of the elongation reaction, 1.75 U Taq HotStar DNA polymerase (Qiagen, Hilden, Germany), 1× PCR reaction buffer, 5 nM dNTP mix, and 4 mM MgCl_2_ (all Qiagen, Hilden, Germany), adjusted with water to 50 µl total volume. The temperature profile was: 95°C for 5 min, followed by 45 cycles of 95°C, 30 sec, 58°C, 30 sec, 72°C, 1 min, and a final elongation step at 72°C for 5 min. PCR reactions were subject to standard gel electrophoreses on a 2% agarose gel using TBE running buffer.

**Table 1:** Overview of alternative primers used in the study.

Sequence Name	Sequence
Boca_end_SspI-1_neu	5′ - CATCATATAACCACTCCTCC - 3′
Sspl 2	5′ - TTTCCTGGGAGTGGTTATGG - 3′
Boca_end_SspI-F	5′ - GGAGGAGTGGTTATATGATG - 3′
Boca_end_NruI-1_neu	5′ - CCGACAGCCCTTGTACATTG - 3′
Nrul 2*	5′ - ACAGC CCCTTGTACATTGTGG - 3′
Boca_end_NruI-F-r	5′- CAATGTACAAGGGCTGTCGG -3′
Boca_end_Nru-2_neu	5′- TTCCTCCTCAATGGACAAGC -3′

The table summarizes the names and sequences of primers used in the study. The primers fit to the reference sequence of HBoV (DQ000496 and should be able to amplify templates of head-to-tail structure or from elongation approaches, provided that no difficult tertiary structures nor mutations are present in the target region. The primers cover a restriction site of SspI or NruI at the terminal regions, respectively. However, the primer binding sites are less conserved as the have solely been described for the DQ000496 sequence so far. All primers are located in the most terminal region possible for primer design.

*The underlined sequence covers a CviKI-1 restriction site that should have appeared in a putative PCR product.

Alternatively to the use of T4-polymerase, the elongation reaction was performed with 50 U DNA polymerase I (Klenow polymerase) (New England Biolabs, Frankfurt a.M., Germany), 1× NEB buffer 2 (New England Biolabs, Frankfurt a.M., Germany), 2 mM dNTP mix, 5 µl of the HBoV DNA eluate, and water adjusted to 25 µl. The following PCR reactions were performed as described above.

Finally, taking into account the single strand nature of the HBoV genome, the eluted HBoV DNA from clinical samples was heat-denaturated for 10 min at 96°C and shock-cooled in an ice water bath. Immediately T4 RNA ligase, which is also able to ligate and self ligate ssDNA, was added and the reaction conditions were set up as recommended by the manufacuter (New England Biolabs, Frankfurt a.M. Germany). Reactions were carried out for for 6 hours. This reaction was also followed by the same PCR as described above with both primers.

### PCR assays for detection of putative head-to-head or tail-to-tail intermediate sequences

**Figure 2:**
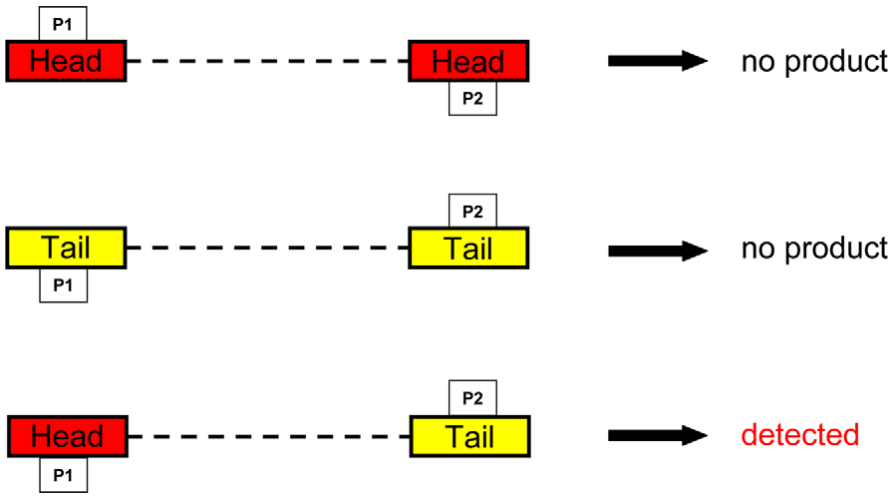
Theoretical structure of concatemeric sequences that could occur during HBoV replication. The boxes indicate the primers that were used for the amplification of the putative concatemeric structures. The broken lines indicate the linking sequence that is dependent on the length of the unknown terminal sequences.

Based on earlier observations and replication models for other parvoviruses it was assumed that head-to-head or tail-to-tail DNA sequences should occur during the HBoV infection cycle [21]. For this reason DNA was extracted from clinical samples as described above. Briefly nasopharyngeal aspirates using the QIAamp DNA extraction kit (Qiagen, Hilden, Germany) followed by PCR using the Qiagen HotStar Taq plus Mastermix (Qiagen, Hilden, Germany). PCRs were performed according to the manufacturer's recommendation and the temperature profile described above. All possible combinations of one head and one tail primer and their reverse complement primers directed against conserved sequences in both regions were tested, respectively (figure 2a); additionally, both the head and the tail primer were used alone in order to re-test the self priming capability of the putative hairpin-end-structures and to test for head-to-head and tail-to-tail structures (table 2). In summary, the primer combinations were: head forward (forward = +); head forward+head reverse (reverse = −); head reverse; head forward+tail forward; head forward+tail reverse; head reverse+tail forward; head reverse+tail reverse; tail forward; tail forward tail reverse; tail reverse (sequences listed in table 3). Any band appearing on agarose gels was gel extracted and sequenced with the respective primers with which the band was amplified. In addition all bands were cloned by TopoTA cloning according to the manufacturer's recommendations into the pCR®4-Topo sequencing vector and sequenced with T3 and T7 primers (all: Invitrogen, Karlsruhe, Germany). Sequencing was performed from both directions and was performed by MWG-Eurofins (Munich, Germany).

**Table 2:** Overview on primers used in this study.

Elongation Primer		
Primer Name	Sequence (5′⇒3′)	Position in DQ000496*
Head (SspI)	GGAGGAGTGGTTATATAGA	74–91
Tail (NruI)	GTGTTACCGTCTCGAACCTAG	5189–5209

Primer for Head/Tail-sequence detection		
head (+)	GTCTCTACAAGTGAGCGGCCT	201–221
head (−)	CAGAGATGTTCACTCGCCGGA	201–221
tail (+)	GTGTTRCCGTCTCGAACCTAG	5188–5208
tail (−)	CACAAYGGCAGAGCTTGGATC	5188–5208

The table summarizes sequences, genome locations, and names of primers used in the study. Primer were used in combinations as indicated in the text and for detection of intermediate DNA sequences as shown in table 2; *GenBank entry DQ000496 covers the first published HBoV isolate and presents the current reference sequence. (+) = forward; (−) = reverse.

**Table 3:** Primer combinations used in the amplification assays for the detection of putative intermediate structures.

P1	P2
head (+)	head (+)
head (+)	head (−)
head (−)	head (−)
head (+)	tail (+)
head (+)	tail (−)
head (−)	tail (−)
head (−)	tail (+)
tail (+)	tail (+)
tail (+)	tail (−)
tail (−)	tail (−)

The primers were used in the indicated combinations in order to test for the occurrence of all possible combinations of head-to-tail, tail-to-tail, or head-to-head combinations irrespective of the genome orientation packaged into viral capsid in a given clinical isolate. P1 and P2 correspond to the scheme in figure 2.

## Results

The terminal sequences of human bocavirus have not yet been described; in analogy to other parvoviruses it is assumed that the HBoV genome is flanked by terminal hairpin like structures, but the sequences of such structures has not been deciphered by now. The hairpin like structures of other parvoviruses are known to initiate DNA replication by a self-priming mechanism [25].

In order to test the hypotheses that HBoV genomes have terminal hairpin-like structures with a self priming capacity elongation assays were performed. Elongation reactions were carried out according to the scheme presented in figure 1a. Initially, based on the assumption that HBoV contains currently unknown palindromic terminal sequences, the self priming capability of HBoV genomes was tested. We assumed that by addition of T7-DNA-polymerase or Klenow-polymerase and nucleotides to isolated HBoV DNA a putative hairpin should have been elongated as hypothesized and shown for the parvovirus replication model, resulting in a large palindromic sequence. Such a palindromic sequence, if synthesized in the elongation assays, can be amplified using a single primer. Consequently, in order to test for successful elongation of this self-priming event, PCR was performed with a single primer of which the binding site was located in the terminal region of the published genomic sequence and of which the complementary binding site should have been newly synthesized by the self-priming elongation reaction. Thereby it has to be noted that primer binding to the very large palindromic structure was kinetically calculated to be preferred to re-annealing of the elongated hairpin. Unfortunately, all of our approaches failed independent of the clinical isolate or the cell culture DNA extract we used, although we performed the approach for both terminal regions of the published genome (figure 1 b and c). As HBoV genomes are in principle packaged in both polarities into viral capsids this assay should have been worked in both directions. Unfortunately, in none of the isolates used for this study any PCR product was generated (figure 1 b and c). Most HBoV isolates have a SspI restriction site at one terminal sequence, and some also carry the NruI restriction site at the other end (such as DQ000495). As not all HBoV isolates carry the NruI restriction site we made use of primer NruI-2 which would have introduced another restriction site into the PCR product that could have been usefully for later cloning of the PCR product. Any band detectable on the agarose gels in figures 1b and 1c was sequenced from both directions, but exclusively human chromosomal DNA was amplified. However, primers used for these experiments would have allowed to clone the PCR products by restriction with NruI (Boca_end_NruI-1_neu), SspI (Boca_end_SspI-1_neu, Sspl 2), or CviKI-1 (NruI-2) (table 1). Due to the fact that no HBoV DNA was amplified, no cloning of PCR products was performed.

It was also impossible to self ligate and amplify the ssDNA genomes of human bocavirus by using T4 RNA ligase that is known to ligate and self-ligate also ssDNA molecules (figure 1 d). The most likely explanation why any of those approaches failed could be less DNA of HBoV, but as long as it is not possible to cultivate HBoV routinely it is difficult to obtain large quantities of virus and one is dependent on high titer clinical specimen. Of note, the assays were not performed with any other member of the parvovirus family as the terminal structures look different between the different viruses (thus it would have generated limited information if the assays would have worked with other parvoviruses) and due to the fact that no further isolates of other human or animal parvoviruses were available for the study. In any case, the PCR followed by the self-ligation assay should have amplified any head-to-tail, head-to-head, or tail-to-tail sequence (figure 1e). Unfortunately, the primers used exclusively matched to the DQ000496 sequence, thus, consequently more conserved primers were necessary. These set of primers were used in the next step of our analyses.

**Figure 3:**
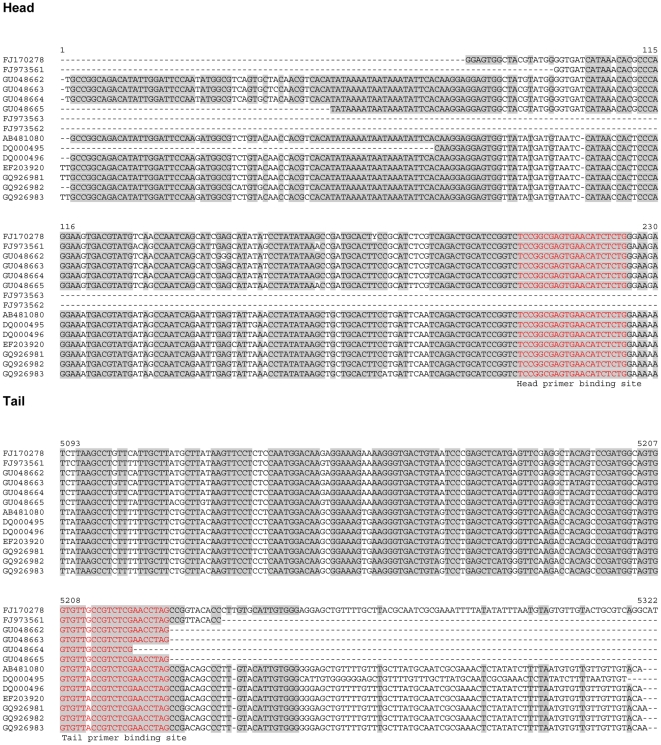
Alignment of head and tail sequences of several published HBoV isolates. Most isolates differ in the length of the published sequences. Red boxes indicate primer binding sites of the head and tail primers used in this study, respectively. Nucleotide numbering is according to the longest isolate sequences used in the alignment.

**Figure 4:**
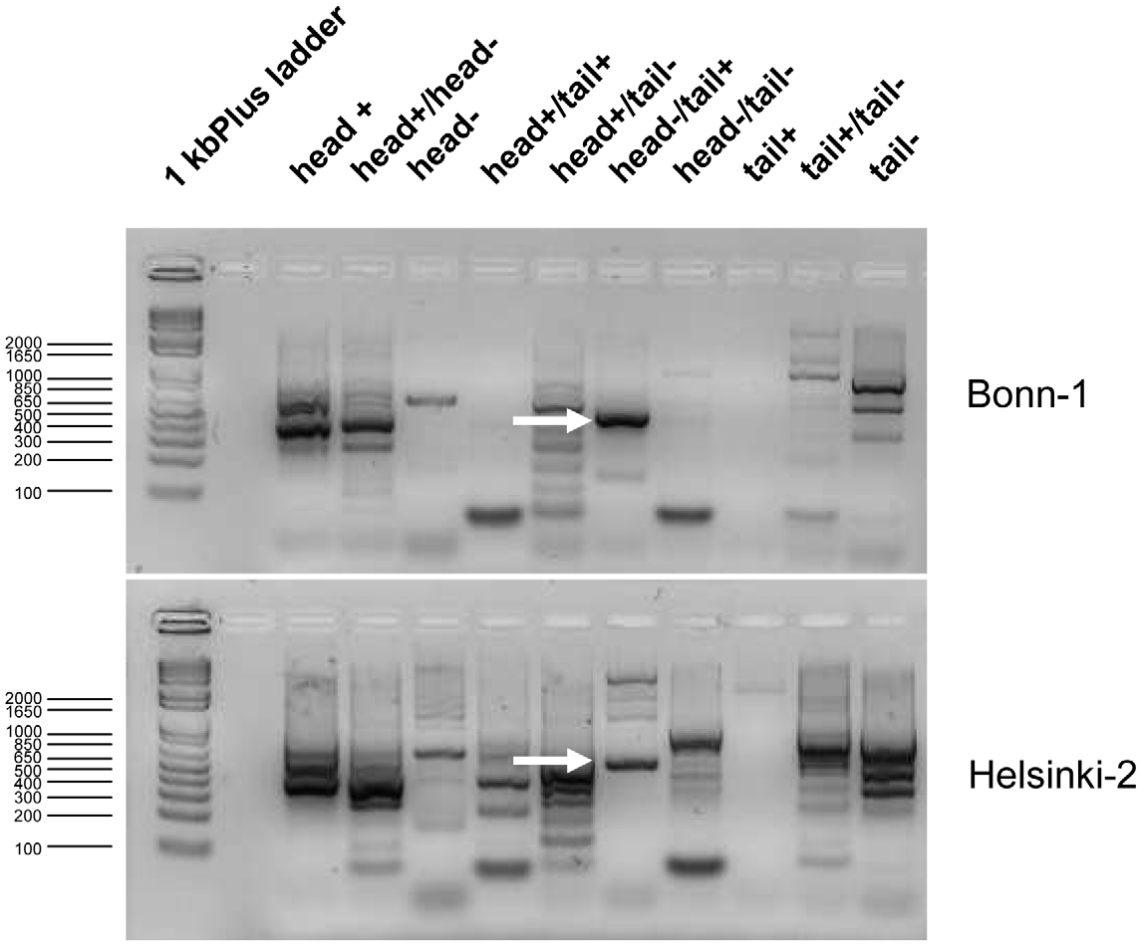
Agarose gel elctrophoreses of PCRs with all possible combinations of head- and tail-primer pairs. Two representative gel pictures are shown. All bands were gel extracted and subject to sequencing and cloning and sequencing from both directions. Arrows indicate the band that contained the HBoV head-to-tail sequences.

**Figure 5:**
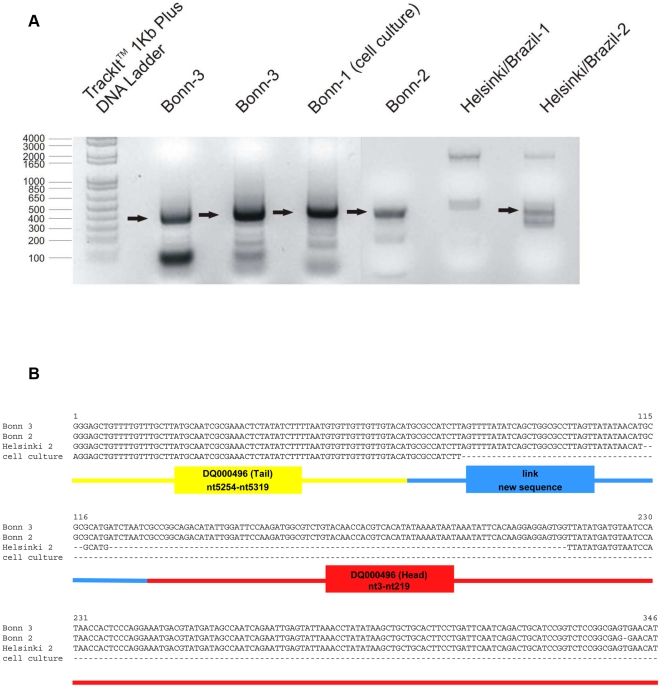
Human bocavirus forma head-to-tail sequences. **a.** ***Agarose gel electrophoreses of head-to-tail PCR from different clinical HBoV-isolates.*** Arrows indicate that the corresponding band was gel-extracted and sequenced. Sequences are aligned in figure 3b. **b.** ***Alignment of Head-to-Tail PCR product from different isolates and cell culture of HBoV.*** All PCR products contained a sequence from the tail of several published clinical isolates that except single point mutations perfectly matches with the prototype sequence of the “Allander strain” DQ000496 (Tail), nt5254–nt5319. This region is followed by a so far unknown sequences, here referred to as linking sequence, which in turn is followed by head sequences conserved in several strains, again perfectly matching to the “Allander strain” DQ000496 (Head), nt3–nt219. The sequence of the PCR amplificates is highly compatible with a classical rolling circle replication (fig. 3c).

In this second step the HBoV isolates were subject to PCRs that should amplify any kind of possible concatemeric sequence postulated for the parvoviral replication cycle (figure 2). PCR reactions were performed with primer combination listed in table 3. The primers were designed from conserved regions we identified by aligning a representative cross-section of published HBoV sequences including the both reference sequences DQ000495 and DQ000496 (figure 3). Those primer pairs theoretically could amplify the concatemeric irrespective of the head-tail orientation in the concatemers and the genome polarity prevalent in the clinical isolate. These PCRs resulted in a couple of bands as shown in figure 4 for three representative isolates. All bands were subject to direct sequencing with their respective PCR-primers and additional cloning and sequencing with standard T3- and T7- primers. Sequencing was performed from both directions. All bands except one were human genomic DNA sequences except those that resulted from the following primer combination head(−)/tail(+): PCR results positive for sequences that contained human bocavirus genome parts were observed solely if PCRs were performed with primer pair forward primer HBoV-Tail 5′- *gtg ttr ccg tct cga acc tag* -3′ and reverse primer HBoV-Head 5′- *cag aga tgt tca ctc gcc gg*a as indicated by arrows in figure 4 and as shown in higher resolution in figure 5a. The PCR product length was approximately 400 bp (figure 5a) and was subsequently sequenced (figure 5b). Sequencing revealed that the PCR products from all isolates consisted of a terminal sequence from the published tail sequences of human bocavirus, followed by a so far unknown region of approximately 50 nucleotides, followed by the head sequence of human bocavirus (figure 5b).

The length of the hitherto unknown sequence was shorter in case of a Brazilian isolate (Helsinki 2) but was identical in two isolates from Bonn, although these two isolates were not epidemiologically related; the sequence obtained from the cell culture did not cover the full head-to-tail amplificate but resembled the German isolates.

## Discussion

The aim of the present study was to identify terminal sequences of the human bocavirus that were postulated to occur beyond the hitherto published genome sequences [3], [4]. Such sequences have been shown to be present in other parvoviruses and are a prerequisite for proper replication of parvoviruses. In order to decipher those putative sequences we made use of the assumption that the terminal sequences mediate a self-priming and trigger the formation of head-to-head or tail-to-tail intermediates [3], [4]. The self-primed elongation approach with T4-DNA polymerase and also with Klenow polymerase followed by PCR with primers located in the very terminal region of published sequences DQ000495 and DQ000496 failed as well as self-ligation of the single stranded genomes from clinical isolates followed by PCR with those primers (figures 1a–e). There are a couple of explanations why those approaches failed; most likely, provided the terminal sequences are present as postulated, self-priming is hindered by secondary structures that cannot be resolved by the used enzymes. It may also be that the sensitivity of the assays was too low for the purpose and that detection of products failed due to the amount of material used in the reaction; this, however, is a general problem that can be solved exclusively by a simple and permanent cell culture model (low budget) or by an exhausting experimental setup of large scale primary cell cultures (very high budget required). Alternatively, the primers used for those pilot experiments were less optimal, as usage of primers from a most conserved region (figure 3) amplified the head-to-tail sequences shown in figure 5. The primers used in the pilot experiments were designed based on the very terminal sequences of two strains ST1 and ST2 and corresponding sequences and changes in this region in other strains remain possible. However, based on the sequence analyses and alignments it appears to be likely that the newly identified part of the HBoV sequence represents a hitherto missing link in the HBoV genome. The identified region was not found completely in any of the published HBoV sequences so far, just 20 bp of the linking region are found in MVC (FJ214110: 5283nt–5302nt) where these nucleotides are part of a palindromic sequence (5′-*atgcgccttagttatataacatt*|*aatgttatataactaaggcgcat*-3′) [8]; anyway, the PCR amplificates were identified to be part of the longest known tail and/or head sequences from published HBoV sequences (see alignments in figure 1 and 2b). It is very unlikely that such a conserved sequence of approximately 50 bases is newly synthesized by the PCR polymerase or derives from recombination procedures during three independent PCR reactions. However, it is possible that during the stay of viral DNA within an infected cell the sequence is inserted and a so far completely unknown mechanism in the replication of parvoviruses, in particular of human bocavirus, takes place. Furthermore, the head-to-tail sequences can result from a PCR reaction on a template with pan-handle structure that is observed for some other parvoviruses [26].

Due to the fact that the clinical and cell culture material that contains human bocavirus DNA is limited it is difficult to analyse the physical properties of the viral genomes and putative hairpin-structures that in turn may be difficult to amplify by PCR. Thus it may have appeared that additional DNA structures could have been amplified by alternative primer combinations and that such putative sequences have been masked by non-specific amplification as observed in our study. In addition it remains possible, that further HBoV sequences were not amplified due to secondary DNA structures that cannot be resolved by PCR protocols.

The present study reveals novel insights into the replication mechanism of human bocavirus. Due to the lack of a routine tissue culture model it remains highly difficult to analyse human bocavirus replication. In this study clinical samples as well as DNA from the so far unique cell culture model containing infected cells that in turn contain replicated human bocavirus DNA were used. We identified head-linker-tail sequences that were not been described so far. These tail-linker-tail sequences could be of different origin: it is possible that these sequences are an artefact of the PCR reactions; this assumption appears unlikely, as within the novel sequence there is also a 20 bp stretch present that is conserved also among the closely related parvovirus MVC. However, if the single stranded HBoV genome forms a panhandle structure that has been described for other parvoviruses [26] it may be that this panhandle structure mimics a covalently closed circular genome that in turn could be misinterpreted as the template for a rolling circle replication; such a rolling circle replication may be another explanation for the head-linker-tail sequences that were identified in the present study. However, the head-linker-tail sequences are incompatible with the rolling hairpin replication [27], [28] mechanism but would fit to the concept of extensive recombination events by polymerase strand exchange as already described for HBoV [29]. The results presented here in this study are highly compatible with the observation that virtually all HBoV-genomes packaged into viral particles are of negative polarity [23]. The nearly exclusive packaging of progeny genomes of a single polarity into newly formed viral particles is a typical feature of a rolling circle replication in its true sense and is supported by the occurrence of head-to-tail concatemeric structures as observed in our analysis. On the other hand, it is also possible that the observed head-linker-tail sequences are a product of recombination events or of a complete novel feature of the replication cycle of HBoV, as the exclusive packaging of negative strands may also be a result of a specific packaging mechanism as described for murine parvoviruses [5]. Another possibility is that the head-linker-tail sequence is part of a dead-end product: The unidirectional parvoviral replication fork observed for dependoviruses like AAV if prone to strand switching, and its products are highly susceptible to recombination, frequently leading to the accumulation of dead-end replication products in infected host tissues [30]. This latter observation by Gao and colleagues would be compatible with the observation by Kapoor and coworkers who postulated high frequency of recombination of human bocavirus [29]. Nevertheless, to test these latter hypothesis that a unidirectional parvoviral replication fork produced dead-end-products more clinical material and optimized replication assays are required. Those replication models may benefit from the newly identified sequence which may be essential for autonomous replication of human bocavirus.

In summary, the present study arises some hypotheses that require further testing. Our study emphasises the need for a simplified cell culture or an animal model to study the overall replication cycle of human bocavirus; until such a model is available, it is crucial to collect more clinical samples in larger amounts in order to enable further analyses on the viral genome structure and replication intermediates.

In the recent past it was frequently assumed that newly detected viruses do behave genetically like their closest relatives without supporting such hypotheses by experimental data. Taking into account the observations of the current study, at least in case of human bocavirus such assumptions seems to be not fully applicable and need further testing.

The presented results are of major interest because they are the next step in understanding the full replication cycle of human bocavirus and thus contribute to the future development of cell culture models and/or replication competent HBoV-vectors that in turn can be used to develop potent disinfectants and drugs against human parvoviruses.
